# Protocol for *ex vivo* culture of neonate mouse ovaries to study early follicle activation

**DOI:** 10.1016/j.xpro.2026.104539

**Published:** 2026-05-08

**Authors:** Julie Feld Madsen, Stine Bundgaard Birkebæk, Karin Lykke-Hartmann

**Affiliations:** 1 Department of Biomedicine, Aarhus University, Aarhus C 8000, Denmark; 2 Department of Clinical Genetics, Aarhus University Hospital, Aarhus N 8200, Denmark

**Keywords:** Cell Biology, Cell culture, Developmental biology, Model Organisms, Molecular Biology

## Abstract

Establishing an *ex vivo* culture of neonatal ovaries enables controlled investigation of early follicle dynamics. Here, we present a protocol for isolating ovaries from neonatal female mice for maintaining intact ovarian architecture by *ex vivo* culture on a membrane allowing access to defined medium. We describe procedures for media preparation, ovary dissection, culture setup, and follicle development by stereomicroscopy and histological assessment. This protocol provides a platform for examining the regulation of the primordial-to-primary transition and growth.

For complete details on the use and execution of this protocol, please refer to Amoushahi et al.^1^

## Highlights

• Instructions for *ex vivo* organ culture of neonatal mouse ovaries
• Steps for H&E-based histology for ovarian tissue
• Guidance on quantification of ovarian follicle developmental stages

## Abstract (editor highlights)

Publisher’s note: Undertaking any experimental protocol requires adherence to local institutional guidelines for laboratory safety and ethics.

## Graphical abstract

**Figure:**
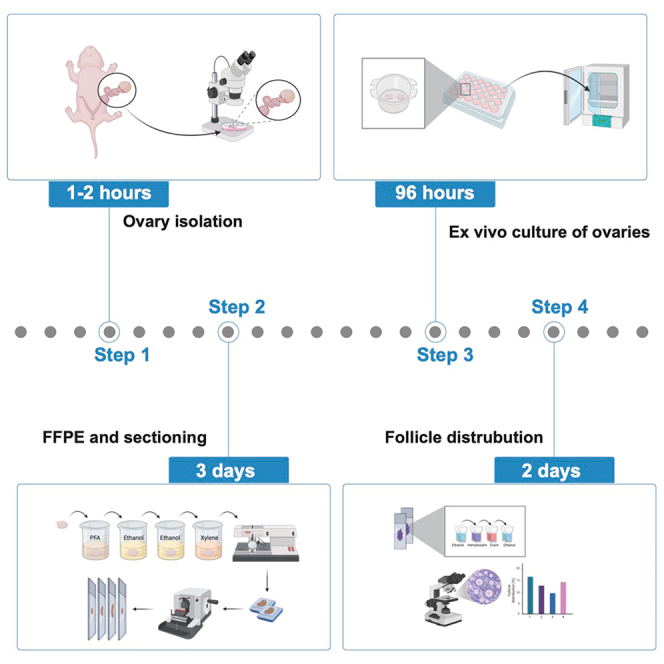

## Before you begin

Folliculogenesis is initiated by the activation of dormant (primordial) follicles into primary follicles, during the primordial-to-primary transition, supported by highly regulated signaling within the ovarian follicles.^2^^,^^3^ Here we describe a method to establish murine ovary *ex vivo* culture, which can subsequently be applied to model early ovarian follicle regulation by modulating specific signaling factors.^1^^,^^4^

Briefly, we introduce ovary dissection from juvenile female mice, the careful and precise dissection needed to maintain intact ovaries. We then describe the step-by-step handling, and *ex vivo* culture including optimal media conditioning. In addition, we add a detailed description for how to fix the ovaries for tissue processing and paraffin embedding before describing the precise procedure for sectioning and H&E staining. Finally, we include a full description for follicle morphology and histological examination.

### Innovation

This protocol advances existing neonatal ovary *ex vivo* culture approaches by optimizing both culture establishment and culture conditions that support preservation of tissue integrity and follicle viability during the *ex vivo* culture period. We compared multiple membrane-systems and identified the configuration that best maintains organ shape and nutrient exchange. The combination of improved *ex vivo* culture steps minimize the extent of apoptosis, which is a common side-effect in ovary organ *ex vivo* cultures. Collectively, these refinements improve reproducibility, reduce variability between culture batches, and provide a more consistent platform for studying early follicle development and regulatory signaling in intact ovaries.

### Institutional permissions

The murine experiments were approved by the Danish national institutional regulations and approved by the Ethics Committee for the use of laboratory animals at Aarhus University.

### Preparation of stock solution

**Timing: 2**–**4 h**

1. Fetal bovine serum (FBS) is thawed at 4°C.
  a. The thawed FBS is gently mixed.
  b. Aliquot into sterile tubes.
  c. Stored at −20°C until use.

***Note:*** Storage time 6–12 months.

2. Insulin-transferrin-selenium (ITS) is aliquoted into sterile tubes.
  a. Stored at 4°C until use.

***Note:*** Storage time 1 month.

3. Penicillin and streptomycin (P/S) are aliquoted into sterile tubes.
  a. Stored at −20°C until use.

***Note:*** Storage time 1 year.

4. Reconstitute the Follicle stimulating hormone (FSH) powder by adding 750 μL of solvent to prepare a 100 mIU/μL solution.
  a. The solution is aliquoted into sterile tubes.
  b. Stored at −20°C until use.

***Note:*** Storage time 6 months.

5. Weigh 0.1 g of sodium selenite (SS) powder.
  a. Dissolve it in 10 mL ddH_2_O to fully homogeneous.
  b. Dilute this solution 1:1000 and achieve final concentration of 10 ng/mL.
  c. Sterilize the solution through a 0.2 μm filter.
  d. Aliquot into sterile tubes.
  e. Store at −20°C until use.

***Note:*** Storage time 1 year.

### Preparation of culture medium for *ex vivo* culture of ovaries

**Timing: 1 h**

6. Prepare medium for organ harvest:
  a. Add 250 μL FBS to 2250 μL α-minimum essential medium (α-MEM).
  b. Mix well.
  c. Pre-equilibrate the medium by incubating at 37°C and 5% CO_2,_ keeping the cap loosely attached, for at least 30 min.
7. Prepare medium for fine dissection of ovaries:
  a. 4445 μL α-MEM is supplemented with 500 μL FBS, 50 μL ITS and 5 μL FSH.
  b. Mix thoroughly and transfer to an embryo dish for dissection.
  c. Pre-equilibrate the medium by incubation at at 37°C and 5% CO_2,_ for at least 30 min.
8. Prepare medium for *ex vivo* culture:
  a. 4390 μL α-MEM is supplemented with 500 μL FBS, 50 μL ITS, 50 μL P/S, 5 μL FSH, and 5 μL sodium selenite.
  b. Mix thoroughly before use.
  c. Pre-equilibrate the media by incubating the 15 mL tubes, with caps loosely attached, at 37°C with 5% CO_2_ for at least 30 min before use.

***Note:*** Culture medium for organ harvest, fine dissection, and *ex vivo* culture must be freshly prepared prior to each medium exchange to ensure optimal nutrient availability and stability of supplemented components.

## Key resources table

**Table:** 

REAGENT or RESOURCE	SOURCE	IDENTIFIER
**Chemicals, peptides, and recombinant proteins**		

Alpha-MEM	Fisher Scientific	Cat# 22571-020 (Gibco)
FBS	Fisher Scientific	Cat# 11550356
Insulin-transferrin-selenium	Fisher Scientific	Cat# 12097549
Pencillin-Streptymycin	Thermo Fisher Scientific	Cat# 15140122
Sodium selenite	Thermo Fischer Scientific	Cat# 41400045
Recombinant follicle-stimulating hormone	Merck	Cat# GONAL-F75IE-IU
Paraformaldehyde	Thermo Fisher Scientific	Cat# J199943-K2
Paraffin	Leica Biosystems	Cat# 39601006
Haematoxylin	Sigma‒Aldrich	Cat# MHS32-1 L
Eosin	Merck	Cat# 17372-87

**Experimental models: Organisms/strains**		

Female mouse: C57BL/6JRj, used from 6 weeks of age	Janvier	C57BL/6JRj
Male mouse: CBA/JRj, used from 6 weeks of age	Janvier	CBA/JRj
Female C57BL/6Jrj x CBA/JRj, used at day 7 or 8		

**Other**		

Corning Transwell 24 well plates; 0.4 μm pore size, 6.5 mm diameter	Corning	3470
24-well plates	Corning	3526
Stereo microscope	Leica	MDG32
CO_2_ incubator	Thermo Scientific	Forma Steri-cycle
Inverted microscope	Leica	DMI4000 B
Processing machine	Leica	TP1020
Embedding machine	Leica	EG1160
Microtome	SLEE	CUT 6062
Dumont #5 Forceps, Biologie Tips, Inox, 11 cm	AgnThos	11252–20
Tokai Hit Thermo Plate	Tokai Hit	TPi-108RH26
Embryo dish		

## Materials and equipment

**Table:** Medium for organ harvest

Reagent	Final concentration	Amount
α-MEM	N/A	2250 μL
FBS	10% (v/v)	250 μL
**Total**	**N/A**	**2500 μL**

**Table:** Medium for fine dissection

Reagent	Final concentration	Amount
α-MEM	N/A	4445 μL
FBS	10% (v/v)	500 μL
ITS	1% (v/v)	50 μL
FSH (100 mIU/μL)	0.1 mIU/μL	5 μL
**Total**	**N/A**	**5000 μL**

**Table:** Medium for ex vivo culture

Reagent	Final concentration	Amount
α-MEM	N/A	4390 μL
FBS	10% (v/v)	500 μL
ITS	1% (v/v)	50 μL
P/S	1% (v/v)	50 μL
FSH (100 mIU/μL)	0.1 mIU/μL	5 μL
Sodium selenite (10 ng/mL)	0.01 ng/μL	5 μL
**Total**	**N/A**	**5000 μL**

***Note:*** Culture medium used for organ harvest, fine dissection, and ex vivo culture should be freshly prepared and pre-warmed to 37 °C prior to each medium exchange. The medium should be equilibrated in an incubator at 37 °C with 5% CO_2_ and used immediately after preparation, without storage, to ensure optimal nutrient availability and stability of supplemented components.

## Step-by-step method details

### Isolation of ovaries

**Timing: 1–2 h**

This section describes the procedure to sacrifice 7–8-days old female mice and isolate their ovaries. The goal is to obtain intact ovarian tissue suitable for subsequent *ex vivo* culture.

**Figure 1:**
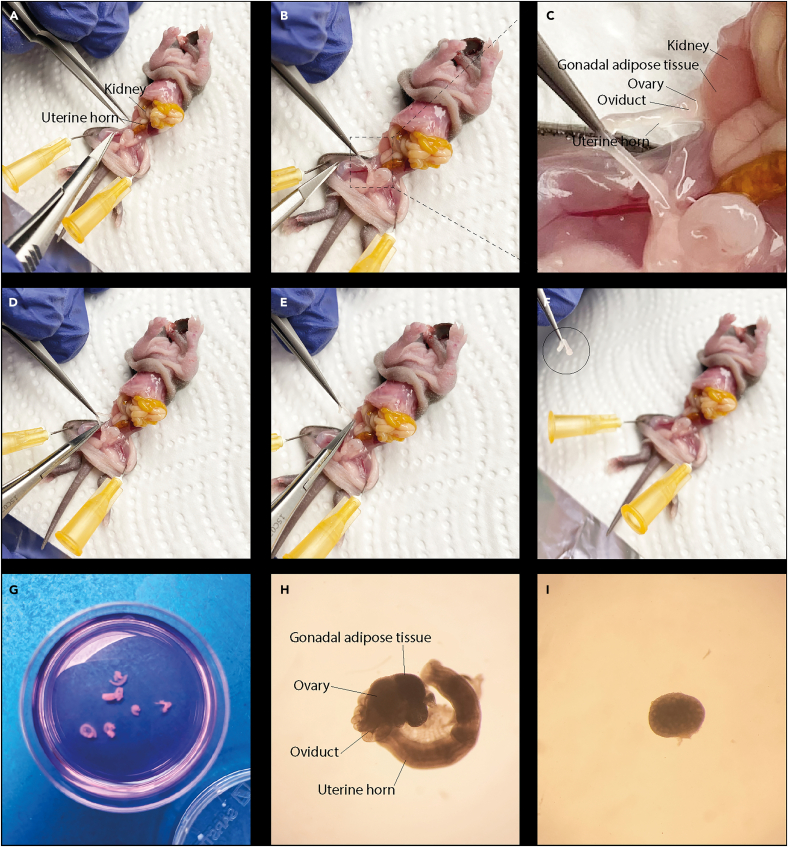
Excision of ovaries from juvenile mice and fine dissection under a stereomicroscope (A) The abdominal cavity is opened, and the gastrointestinal tract is gently displaced to expose the dorsal abdominal region and facilitate localization of the kidneys and uterine horns. (B) Using fine forceps, the uterine horn is carefully lifted to aid visualization of the ovary and associated reproductive structures. (C) Close-up view showing the kidney, gonadal adipose tissue, ovary, oviduct, and uterine horn. (D) The first incision is made in the uterine horn just distal to the ovary using surgical scissors. (E) A second incision is made proximal to the ovary, near the lower pole of the kidney. (F) The ovary is released together (identified in the circle) with its surrounding gonadal adipose tissue. (G) Collected ovaries are transferred to pre-warm medium on a heated stage. (H) Under the stereomicroscope, the excised ovary is visualized with attached gonadal adipose tissue, oviduct, and uterine horn prior to fine needle dissection. A calibrated image of the same ovary, including a scale bar, is shown in panel Figure 2J for reference. (I) The fully dissected ovary with surrounding tissues removed. A calibrated image of the same ovary, including a scale bar, is shown in panel Figure 2J for reference.
7. Carefully release each ovary from surrounding tissue, ensuring the ovary remains intact (Figures 1D–1F).
8. Immediately transfer the ovaries to the pre-wared organ harvest medium maintained at 37°C (Figure 1G).
9. Processed directly to the laboratory and begin the dissection without delay.

***Note:*** It is important that this step is performed promptly to preserve tissue integrity.

1. Transfer the pre-warmed organ harvest medium into an embryo dish and place it on a 37°C heated stage to maintain temperature during ovary isolation.
2. Postnatal day 7–8 female mice are euthanized by decapitation in accordance with approved institutional animal care guidelines.
3. Place the mouse in a supine position.
4. Clean the abdominal area with 70% ethanol.
5. Gently separate the abdominal skin and underlying peritoneal layer by applying opposing lateral tension with both hands to expose the abdominal cavity.
6. Identify the ovaries adjacent to the kidneys and connected to the uterine horns (Figures 1A–1C).

10. In the laboratory, the ovaries are transferred to the warm fine dissection medium using forceps.

**CRITICAL:** Handle the ovaries gently to avoid compressing or damaging the tissue.

11. Gently remove surrounding connective tissue and release the ovary from the ovarian bursa.
  a. Release the ovary from the ovarian bursa by carefully teasing the tissue apart under the stereomicroscope using fine needles.
  b. Stabilize the ovary with one needle while gradually separating attachment with the other, taking care not to puncture or compress the ovarian tissue.
  c. Continue until the ovary is fully free from the bursa and connective tissue, preserving the integrity of the ovarian capsule (Figures 1H and 1I).

***Note:*** Removing the bursa and surrounding connective tissue is critical to ensure proper penetration and uptake of the culture medium by the ovary.

12. Finally, the isolated ovaries are kept in the fine dissection medium and placed in the incubator at 37°C and 5% CO_2_ while preparing next step.

### Seeding ovaries onto membranes for *ex vivo* culture

**Timing: 30 min**

This section detail how to place the freshly isolated ovaries onto culture membranes, ensuring proper orientation and support.

**Figure 2:**
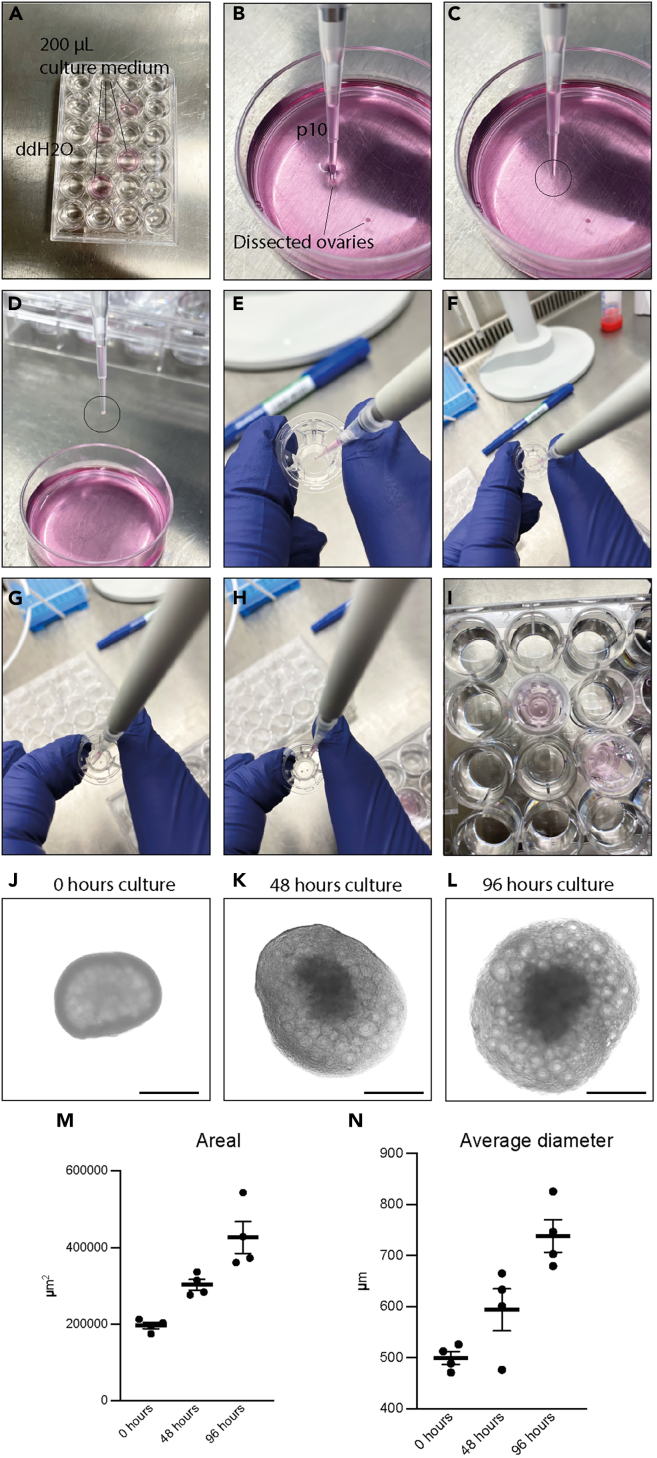
Seeding of ovaries onto membranes and *ex vivo* growth of ovary for 96 h (A) Overview of the 24-well plate setup. Four wells contain 200 μL culture medium, while the remaining wells are filled with water to maintain humidity. Additionally, water is placed between wells to prevent evaporation. (B) Embryo dish containing culture medium used to collect dissected ovaries. The tip of a P10 pipette is shown as the tool used for transferring ovaries. (C) An ovary positioned at the opening of a P10 pipette tip prior to placement. (D) Removal of excess medium from the pipette tip, demonstrating that the ovary adheres to the edge of the tip without being aspirated into the lumen. (E) Membrane insert into which the ovary will be positioned. (F) Placement of the first ovary onto the membrane surface. (G) Positioning of the second ovary on the same membrane. (H) Membrane inserts carrying ovaries prior to culture. (I) Insert placed into a well of the 24-well plate containing culture medium, ensuring that the membrane is in minimal contact with the liquid phase. (J) Photomicrograph of an ovary immediately after placement on the membrane insert. Scale bar: 500 μm. (K) Photomicrograph of ovary after 48 h of culture, showing increased size due to follicular growth and expansion of the ovarian cortex. Scale bar: 500 μm. (L) Photomicrograph of the ovary after 96 h of culture, demonstrating further enlargement consistent with ongoing follicular development. Scale bar: 500 μm. (M) Ovary area measured from photomicrograph. Data are presented as mean ± SEM (n=4 independent ovaries per group). (N) Average ovary diameter, calculated as the mean of horizontal and vertical measurements. Data are presented as mean ± SEM (n=4 independent ovaries per group).

***Note:*** Limit each 24-well plate to a maximum of six inserts, as exceeding this number may reduce humidity.

13. Add 200 μL of culture medium to each well designated for inserts.
14. Fill the remaining wells and inter-well spaces with sterile ddH_2_O to maintain adequate humidity (Figure 2A).

15. Transfer two ovaries onto each membrane in the insert using a P10 pipette.
  a. First, aspirate a small volume of culture medium from the dish containing the dissected ovaries.
  b. Then carefully draw a single ovary to the pipette tip (Figures 2B–2D).
  c. Gently dispense the medium onto the insert, allowing the ovary to settle onto the membrane - without direct contact - from the pipette.
  d. Remove any excess medium from the insert (Figures 2E–2H).

***Note:*** Place the two ovaries near the center of the insert, avoiding placement too close to the edges or too close to each other to ensure uniform medium exposure and optimal culture condition.

16. Position the insert to float on the culture medium, ensuring light contact without submersion (Figure 2I).

**CRITICAL:** Confirm that the membrane is in contact with the medium. If contact is incomplete, gently tap the bottom of the plate to establish proper medium contact.

17. Place the culture plates in a humidified incubator at 37°C with 5% CO_2_ for 96 h, and 70 μL of culture medium is replaced every other day with fresh culture medium.

***Note:*** Prepare fresh medium for every exchange to maintain optimal culture conditions.

***Note:*** The culture period can be adjusted depending on experimental goals, ranging from a few hours up to seven days.

18. Photomicrographs of the ovaries can be taken on an inverted microscope to follow the growth of the follicles within the ovary (Figures 2J–2L).

### Formalin fixation and paraffin embedding of ovaries

**Timing: 2 days**

This section explains how to fix the ovaries after the culture period and embed them in paraffin. The purpose is to preserve tissue architecture for downstream histological analysis.

**Figure 3:**
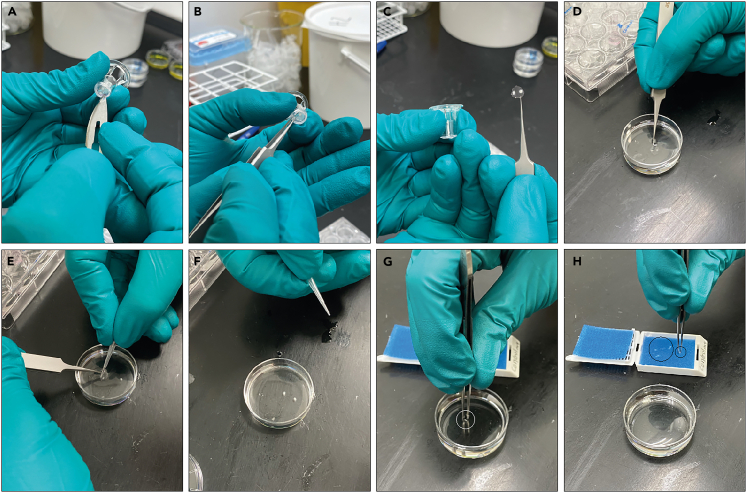
Fixation and preparation for processing of *ex vivo* cultured ovaries (A) The insert membrane containing the ovaries is loosened by carefully cutting around the edge with a scalpel, leaving a small uncut section to keep the membrane attached to the insert. (B) Using forceps, the membrane is detached from the insert. (C) The membrane with the two ovaries is now detached from the insert. (D) The insert is transferred to a Petri dish containing 4% PFA with the ovaries facing upwards. (E) Using a p10 pipette tip, the ovaries are gently loosened from the insert. (F) The ovaries are fixated in 4% PFA at 4^o^C overnight. (G) The next day, the ovaries are gently removed from the PFA using forceps. (H) The ovaries are placed on biopsy pads for processing. Black circles indicate the ovaries.
22. Place the membrane with the ovaries into the PFA solution.
  a. Gently agitate to release the ovaries; if necessary, use fine forceps to carefully free the ovaries, taking care to preserve the ovarian edges where follicles reside (Figures 3D–3F).
23. Incubate ovaries in PFA at 4°C overnight (16–20 h) to complete fixation.
24. The next day, prepare embedding cassettes with two biopsy pads each.
  a. Label each cassette with the experimental group, date, and number of ovaries using a pencil. Each cassette accommodates 4–5 ovaries.
25. Transfer ovaries onto the biopsy pads using fine forceps.
  a. Handle the ovaries gently, using only the tension between the forceps tips to capture the tissue without compression (Figures 3G and 3H).

***Note:*** If ovaries adhere to the forceps, apply a small drop of PFA on the sponge to facilitate release.

19. At the end of the culture period, prepare one petri dish (2–3 cm diameter) per experiment. Fill each dish with 4% paraformaldehyde (PFA) in a fume hood.
20. Transfer culture plates to the fume hood.
21. Carefully remove each insert and using a scalpel.
  a. Detach the membrane from the insert by cutting from underneath, releasing approximately three-quarters of the membrane.
  b. Then remove the insert completely with forceps (Figures 3A–3C).

26. Place a second biopsy pad on top of the ovaries, creating a sandwich, and close the cassette.

***Note:*** Position the cassettes into the tissue processor and process tissues according to the following program (Table 1):

***Note:*** As an alternative to automated tissue processing, samples can be manually processed by sequential incubation in increasing concentrations of ethanol for dehydration, followed by clearing in xylene and infiltration with molten paraffin.

**Figure 4:**
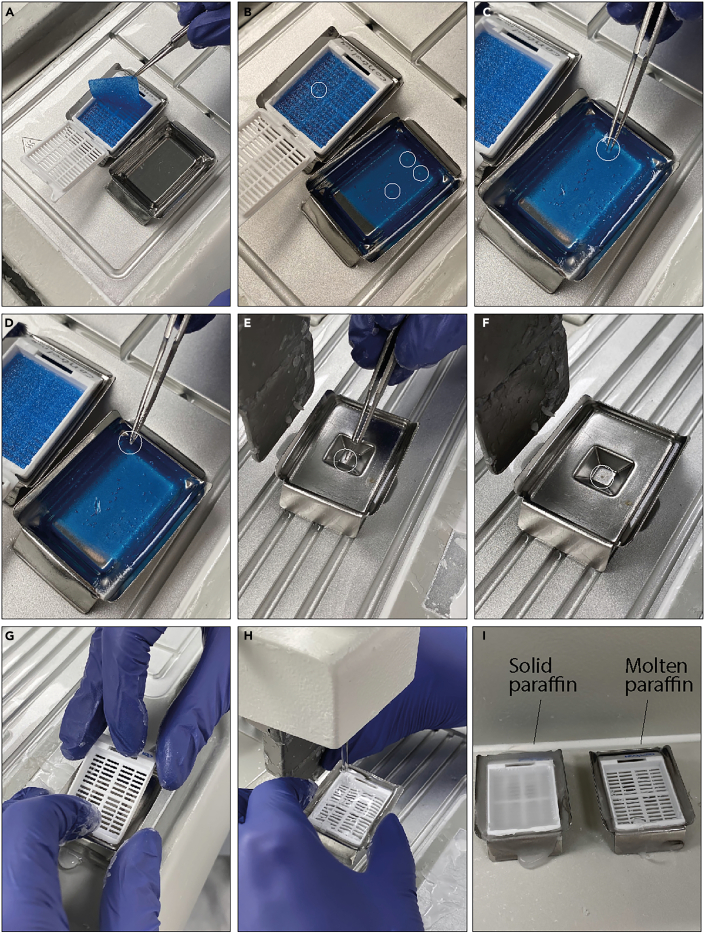
Embedding of *ex vivo* cultured ovaries (A) The cassette from processing is placed in a mold with 60^o^C warm paraffin. (B) The biopsy pads are placed in the molds with the ovaries facing upwards. The ovaries are marked with circles. (C) Using forceps with a small amount of solid paraffin at the tip, the ovary are transferred by sticking the ovary to the tip. (D) The ovary is transferred to the mold. (E) The forceps are placed in the mold filled with molten paraffin. Allow the ovary to detach from the forceps by waiting for the paraffin to molt, and the ovary will sink to the bottom of the mold. (F) The ovary is placed in the middle of the mold. (G) Place a labeled cassette gently on top of the mold containing the ovary. (H) Fill additional paraffin in the cassette. (I) Let the paraffine solidify for at least 30 min.
  b. Place a cassette (labelled with the experimental group, date, and number of ovaries) on top of the mold.
  c. Fill the mold with additional paraffin needed.
  d. Allow to solidify on a cold plate for at least 30 min (Figures 4G–4I).
  e. Store paraffin-embedded ovaries at 4°C for at least 24 h before sectioning them.

27. After processing, the ovaries are embedded into paraffin.
  a. Transferring the ovaries into molds containing molten paraffin. The ovaries are positioned centrally in the molds (Figures 4A–4F).
    ***Note:*** Avoid compressing the tissue; gentle handling is sufficient.

**Table 1:** Processing program

Step	Reagent	Time (min)
Dehydration	70% EtOH	20
	80% EtOH	20
	90% EtOH	20
	100% EtOH	20
	100% EtOH	20
Clearence	Xylene substitute	5
Infiltration	Paraffin 60°C	30
	Paraffin 60°C	30

### Sectioning and H&E staining

**Timing: 1 day**

This section describes how to section the paraffin-embedded ovaries using a microtome. This goal is to obtain thin, uniform slices suitable for H&E staining.

***Note:*** Duration may increase with sample number

**Figure 5:**
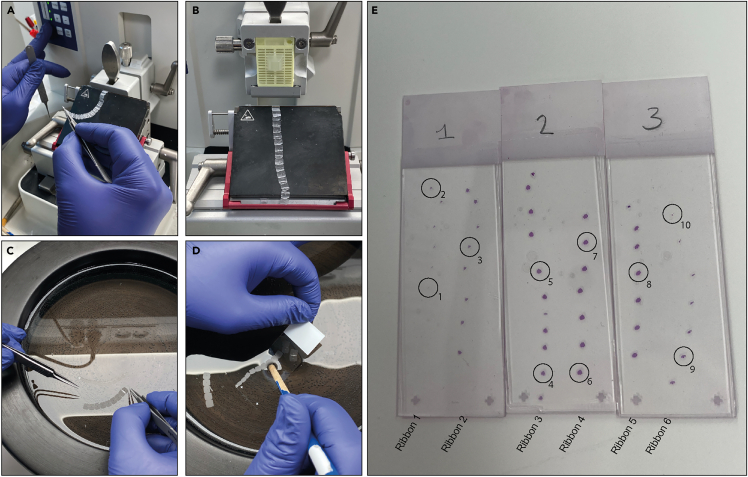
Sectioning All sections are collected sequentially to preserve section order (A) Initiation of a paraffin ribbon during microtomy, showing the continuity of serial sections. (B) Complete ribbon positioned on the microtome. (C) Transfer of the ribbon onto a warm water bath using fine forceps to flatten and prepare sections for mounting. (D) Sequential transfer of ribbon of sections from the water bath onto a microscope slide, emphasizing maintenance of correct section order. (E) An ovary was serially sectioned and mounted in the correct order across three slides and subsequently stained with H and E. Ribbons 1-6 indicate the sequential order of sectioning and slide mounting. Every fifth section is marked with a circle and numbered in the correct order; these sections were selected for follicle counting.
  i. Maintain serial order of sections.
    **CRITICAL:** As every fifth section will be used for subsequent analysis it is critical to maintain the sections in the correct order. For optimal organization, prepare ribbons consisting of 8–9 consecutive sections. Leave the final section attached to the blade during transfer to prevent rolling and loss of tissue integrity.
  j. Float ribbons on a 37°C water bath to allow full expansion of the paraffin and tissue.
  k. Transfer sections to pre-labeled slides using fine needle and remember to maintain correct orientation (Figures 5C and 5D).
  l. Continue sectioning until the entire ovary has been processed.
    ***Note:*** The total number of sections per ovary varies depending on culture duration and treatment conditions. As a general guideline after 96 h of culture, it is estimated to generate 40–60 sections per ovary.

28. Sectioning
  a. Fill the water bath with distilled water and set the temperature to 37°C.
  b. Prepared glass slides labeled with group name, ovary number, and date.
  c. Mount the paraffin block containing the ovaries in the microtome.
  d. Install a new blade.
  e. Set the section thickness to 5 μm.
  f. Adjust the knife position close to the paraffin block without making contact.
  g. Trim the block until tissue sections appear.
  h. Once tissue is exposed, reduce the cutting speed to a moderate level to obtain continuous ribbons (Figures 5A and 5B).
    **CRITICAL:** Collect all sections containing ovarian tissue, from the initial to the final section, while carefully minimizing section loss to preserve complete tissue representation.

29. Hematoxylin-Eosin staining (Table 2).
  a. Place the slides with sections in a rack.
  b. Incubate in a pre-warmed oven at 60°C to melt the paraffin. All subsequent H&E steps are summarized as a list end of section.
  c. Deparaffinize the slides by immerse slide in two baths of xylene (15 min each).
  d. Rehydrate the slides by immerse slide through a graded ethanol series.
    i. Immerse slides in 100% ethanol three times, 2 min each.
    ii. Immerse slide in 96% ethanol for 2 min.
    iii. Immerse slides in 70% ethanol for 2 min.
  e. Rinse in distilled water for 2 min.
  f. Transfer slides to hematoxylin.
    ***Note:*** Determine the optimal staining time by testing a single slide first, staring with a brief exposure (e.g.: 10 s for fresh hematoxylin), and adjust as needed based on microscopic evaluation.
  g. Rinse all slides under running tap water for 5 min.
  h. Transfer the slides into eosin.
    ***Note:*** Determine the optimal staining duration by testing a single slide first and adjusting based on microscopic evaluation before processing the remaining slides.
  i. Dehydrate slides by transferring them sequentially through a graded series of ethanol.
    i. 96% ethanol (20 times up and down).
    ii. 96% ethanol for 2 min,
    iii. 100% ethanol two times, for two minutes each.
  j. Immerse slides in xylene for 2 min, followed by an additional bath for 30 min to fully clear the tissue.

**Table 2:** H and E steps

Step	Solution/Bath	Duration	Notes
1	Xylene 1	15 min	–
2	Xylene 2	15 min	–
3	Ethanol 100% 1	2 min	–
4	Ethanol 100% 2	2 min	–
5	Ethanol 100% 3	2 min	–
6	Ethanol 96%	2 min	–
7	Ethanol 70%	2 min	–
8	Distilled water	2 min	–
9	Hematoxylin	Variable	Test one slide first to determine optimal time; then stain remaining slides
10	Running tap water	5 min	–
11	Eosin	Variable	Test one slide first to determine optimal time; then stain remaining slides
12	Ethanol 96%	2 min	Agitate rack up and down 20 ×
13	Ethanol 96%	2 min	–
14	Ethanol 100% 1	2 min	–
15	Ethanol 100% 2	2 min	–
16	Xylene 1	2 min	–
17	Xylene 2	30 min	The slides can stay in xylene for an extended amount of time

30. Mount slides using three drops of mounting medium on the slides.
31. Add a cover glass.
32. Allow to dry for at least 24 h before handling or storage.

### Histological analysis and follicle classification

**Timing: 1 day**

This section outlines how to perform H&E staining and classify pre-antral follicle stages based on the morphology. The aim is to quantitative assess follicle development after culture.

***Note:*** Duration may increase with sample number

33. Select every 5^th^ ovarian section for analysis (Figure 5E).
34. Count only follicles with a clearly visible oocyte nucleus to avoid double counting the same follicle.

***Note:*** Follicle counting can be performed using a light microscope with sufficient magnification to clearly distinguish follicular morphology. Alternatively, digitized images obtained from slide scanning may also be used for analysis. Regardless of the method, the magnification or resolution must be sufficiently high to clearly identify the morphological characteristics of each follicle type.

**Figure 6:**
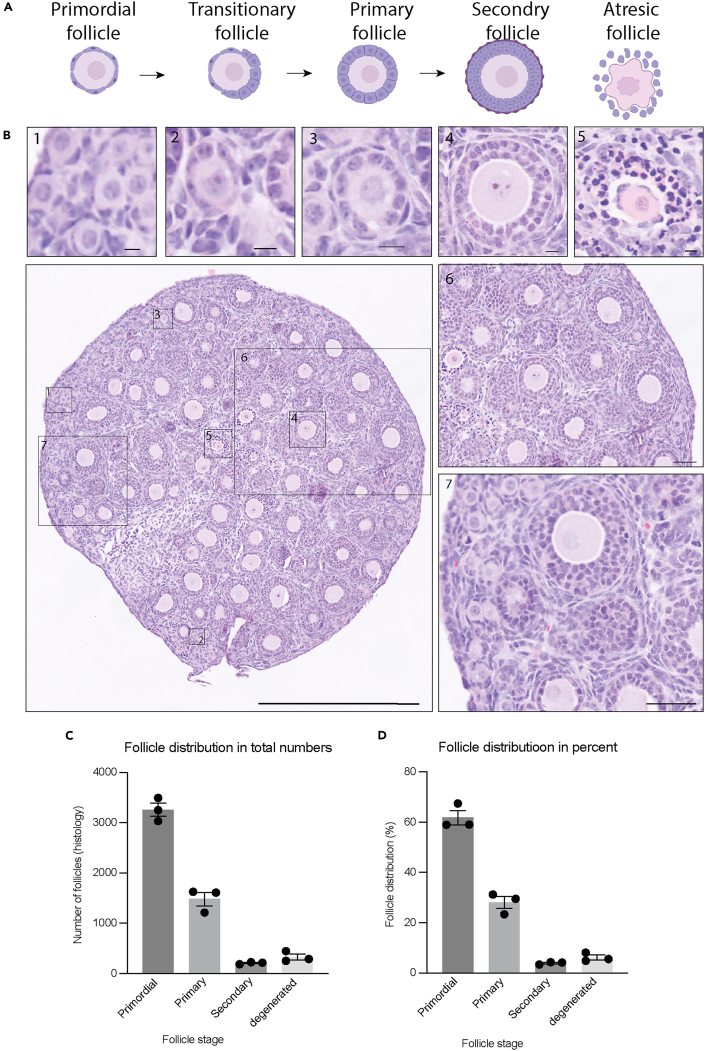
Overview of *ex vivo* ovary culture and follicle classification (A) Schematic illustration of pre-antral follicle classes in cultured ovaries, including primordial, transitional, primary, secondary, and atretic follicles. The animation highlights the defining morphological criteria used to discriminate between stages. (B) Representative H and E-stained section of an ovary following *ex vivo* culture. Scalebar 500 μm. Boxes positioned above the main image denote the location of follicles at specific developmental stages (1: primordial; 2: transitional, 3; primary, 4; secondary, 5; atretic). Scalebar: 10 μm. Corresponding close-up images displayed to the right of the main section provide detailed visualization of detailed visualization of local tissue architecture and follicular morphology. Scale bar 50 μm. (C) Follicular distribution in total numbers. (D) Follicular distribution in percentage.

35. Classify follicles based on morphology:

***Note:*** Primordial follicles: oocyte surrounded by a single layer of flattened pre-granulosa cells (Figures 6A and 6B).

***Note:*** Primary follicles: Oocyte surrounded by one layer of cuboidal granulosa cells or a mixture of flattened and cuboidal granulosa cells (thus including transitional follicles) (Figures 6A and 6B).

***Note:*** Secondary follicles: oocyte surrounded by two or more layers of cuboidal granulosa cells (Figures 6A and 6B).

***Note:*** Degenerated follicles: follicles exhibiting shrunken ooplasm, pyknotic oocyte nucleus, or disorganized granulosa cells (Figures 6A and 6B).

***Note:*** Follicle counts can be expressed either as the estimated total number per ovary, calculated by multiplying the counted follicles by the interval between sections, or as a percentage of the total follicles (Figures 6C and 6D).

## Expected outcomes

This protocol enables *ex vivo* culture of intact murine ovaries, preserving intra-ovarian signaling networks and endogenous regulators, thereby closely recapitulating the physiological microenvironment and making it a valuable tool to study primordial-to-primary follicle transition.^5^^,^^6^^,^^7^ This platform enables pharmacological interrogation, providing a robust system for mechanistic studies of primordial to primary follicle transition in a controlled environment. Based on our observations, the vast majority of ovaries remain viable after 96 h of *ex vivo* culture. Survival is consistently high, provided the tissue has not been subjected to excessive mechanical stress during isolation or handling. Ovaries that experience mechanical damage typically show impaired growth, whereas intact samples maintain continued follicular development. When added to the ovarian culture medium, pharmacological treatments can be used to assess their effects on the transition from primordial to primary follicles through histological analysis and follicle quantification. Resulting data analysis, including follicle counts can be subjected to statistical analysis, such as a *t* test, one-way or two-way ANOVA, depending on the experimental set up, enabling comparison of different experimental groups. Moreover, cultured ovaries can be subjected to Western blotting analysis, RNA sequencing and other biochemical parameters (e.g., Reactive Oxidative Stress (ROS) assay), relevant for the specific experiment.^1^^,^^4^^,^^8^^,^^9^

Furthermore, secondary follicles can be isolated and matured *ex vivo* to metaphase II oocytes, which can be fertilized and transferred into pseudo-pregnant recipients to produce viable offspring, enabling functional assessment of oocyte competence, as demonstrated.^1^^,^^4^^,^^8^

## Limitations

The *ex vivo* culture of murine ovaries enables the preservation of intra-ovarian signaling and follicle activation. However, this system can only partially replicate the complexity and *in vivo* conditions. Notably, dynamic interactions and intricate physiological responses present in the living organism are absent, including endocrine signaling from the hypothalamic-pituitary-ovarian axis and reproductive tract. Additionally, culturing the entire ovary may limit oxygen diffusion to the medulla, potentially resulting in reduced oxygen availability, impaired cellular function, compromised follicle growth, and decreased survival in the center of the ovary.

A key limitation of this protocol is that whole ovary culture reflects follicle activation at the level of the entire organ, without allowing direct distinction between responses in oocytes versus surrounding granulosa cells. As a result, mechanistic insights into cell type–specific signaling within individual follicles cannot be obtained from this protocol. Moreover, each ovary contains a heterogeneous pool of follicles at different developmental stages, including primordial, primary, and secondary follicles, which may complicate the interpretation of stage-specific effects in mechanistic assays.

Additionally, when using *ex vivo* ovary culture, a small amount of tissue is obtained. This restricts the range of downstream assays that can be performed. For example, many activity-based or biochemical assays require larger quantities of material than a single ovary provides, making them unfeasible unless an impractically large number of ovaries are pooled together after end culture. Therefore, the tissue requirements of planned analyses should be considered when setting up *ex vivo* culture.

## Troubleshooting

### Problem 1

After 96 h of *ex vivo* culture, some regions of the ovary show poor growth, visible as clearly defined edges rather than the expected smooth tissue expansion.

### Potential solution

When placing ovaries in culture (step 15), ensure that paired ovaries are not positioned too close to each other, as proximity can inhibit growth on the adjacent sides. Similarly, avoid placing ovaries too close to the well edges, as this can also restrict tissue expansion and lead to uneven growth.

### Problem 2

During microtome sectioning, the ovary detaches completely from the paraffin block, leaving an empty cavity in the paraffin and resulting in loss of the embedded tissue.

### Potential solution

Ensure that all processing reagents are fresh and free of precipitates. Furthermore, ensure that the tissue processor remains completely stationary during processing, as mechanical movement can cause the reagents to be forced through the tissue, leading to tissue damage and subsequent detachment from the paraffin block doing sectioning (Step 27).

### Problem 3

During histological analysis, follicles are difficult to clearly distinguish and classify.

### Potential solution

Use strong hematoxylin to visualize nuclei and light eosin to keep follicles distinguishable for easier counting (Step 30).

### Problem 4

Histological analysis of the ovaries exhibits structural damage, especially along the edges, resulting in compromised tissue integrity.

### Potential solution

Gentle handling during processing is essential to minimize tissue damage (Step 25). Ovaries should only be guided lightly with forceps and never compressed and contact with the tissue should be kept to a minimum due to the fragility of the edges. Despite careful technique, some samples will certainly be compromised, which should be accounted for when planning the number of biological replicates.

## Resource availability

### Lead contact

Further information and requests for resources and reagents should be directed to and will be fulfilled by the lead contact, Karin Lykke-Hartmann (kly@biomed.au.dk).

### Technical contact

Technical questions on executing this protocol should be directed to and will be answered by the technical contact, Julie Feld Madsen (jfm@biomed.au.dk).

### Materials availability

This study did not generate new unique reagents.

### Data and code availability

The published article^1^ includes all datasets generated during this study. The bulk RNA sequencing data reported in the paper have been submitted to the GEO database under accession number GEO: GSE230259 (https://www.ncbi.nlm.nih.gov/geo/query/acc.cgi?acc=GSE230259).

## Acknowledgments

We are very grateful to the reproductive biology laboratory at the Department of Biomedicine, Aarhus University, for discussions and help. The work is supported by grants from the 10.13039/501100009708Novo Nordisk Foundation (NNF16OC0022480 and NNF17OC0026820 to K.L.-H.), the 10.13039/501100004954Augustinus Foundation (17-4844 to K.L.-H.), the 10.13039/501100003035Aase and Ejnar Danielsen Fund (18-10-0470 to K.L.-H.), the 10.13039/501100002739Aarhus University Research Fund (AUFF-E-2020-9-11), 10.13039/501100006309Riisfort Fonden (to J.F.M.). Finally, the graphical abstract and follicle animations in Figure 6 were created with BioRender.com.

## Author contributions

J.F.M. and K.L.-H. developed this protocol, and J.F.M., S.B.B., and K.L.-H. wrote the manuscript.

## Declaration of interests

The authors declare no competing interests.

## Declaration of generative AI and AI-assisted technologies in the writing process

During the preparation of this work, the authors used AI-based tools to assist with language editing and grammar. The authors take full responsibility for the content.
